# Persistent increase in cardiac troponin I in Fabry disease: a case report

**DOI:** 10.1186/1471-2261-11-6

**Published:** 2011-01-31

**Authors:** Christian Tanislav, Andreas Feustel, Wolfgang Franzen, Oliver Wüsten, Christian Schneider, Frank Reichenberger, Arndt Rolfs, Nicole Sieweke

**Affiliations:** 1 Department of Neurology, Justus Liebig University, Giessen, Germany; 2 Department of Nephrology, Justus Liebig University, Giessen, Germany; 3 Department of Cardiology, Justus Liebig University, Giessen, Germany; 4 Department of Radiology, Justus Liebig University, Giessen, Germany; 5 Department of Pulmonology, Justus Liebig University, Giessen, Germany; 6 Albrecht-Kossel Institute for Neuroregeneration, University of Rostock, Rostock, Germany

## Abstract

**Background:**

Hypertrophic cardiomyopathy is a frequent manifestation in Fabry disease (FD) - an X-linked lysosomal storage disorder caused by reduced activity of the enzyme α-galactosidase A. In FD an elevation of specific cardiac biomarkers, such as cardiac troponin I (cTNI) has been reported in case of clinical manifestation suggestive of myocardial ischemia. In diagnosing acute myocardial infarction cTNI is considered the most reliable parameter.

**Case Presentation:**

In the referred case we present a 59 years old female patient with the diagnosis of FD presenting with persistently increased cTNI level (lowest value 0.46 ng/ml, highest value 0.69 ng/ml; normal range <0.05 ng/ml) over a period of 5 months lacking cardiac clinical signs. Since renal insufficiency did not explain the degree of cTNI elevation, this was interpreted as a result of cardiac involvement in FD. Cardiac MRI showed marked left ventricular hypertrophy and focal late Gadolinium enhancement.

**Conclusions:**

Our case report demonstrates a persistent cTNI release in FD with cardiac involvement. Proving the persistence in a symptom free interval, it might be related to a direct damage of myocytes. In FD cTNI could serve as a beneficial long term parameter providing new perspectives for screening strategies.

## Background

Fabry disease (FD) is an X-linked lysosomal disorder caused by reduced activity of the enzyme α-galactosidase A, leading to excessive deposition of glycosphingolipids in the vascular endothelium and tissues throughout the body [1,2]. In the heart, glycosphingolipids deposition causes progressive left ventricular hypertrophy that mimics the morphological and clinical characteristics of hypertrophic cardiomyopathy [1,3]. In FD an elevation of specific cardiac biomarkers, such as cardiac troponin I (cTNI) has been reported in case of clinical manifestation suggestive of myocardial ischemia [4-6]. In diagnosing acute myocardial infarction cTNI is considered the most reliable parameter [7,8]. However, in different disorders a cTNI elevation could be observed without any obvious cardiac involvement and even without clinical sings [9-15]. It is so far unknown whether increased cTNI levels in FD occur in clinically silent intervals. Long term profiles of cTNI in FD patients with cardiac involvement have not yet been reported.

We report on a patient with cardiac involvement in FD presenting with persistently elevated cTNI over a period of 5 months, lacking of cardiac clinical manifestations.

## Case Presentation

In a 57 years old female patient a screening for FD has been carried out due to painful neuropathy. A genetic analysis confirmed the diagnosis of FD (c.424T > C, [C142R]) [16]. An extensive subsequent work-up revealed an advanced organ manifestation including the eyes, kidneys, peripheral and central nervous system and the heart. Consequently an enzyme replacement therapy (ERT) was established.

**Figure 1:**
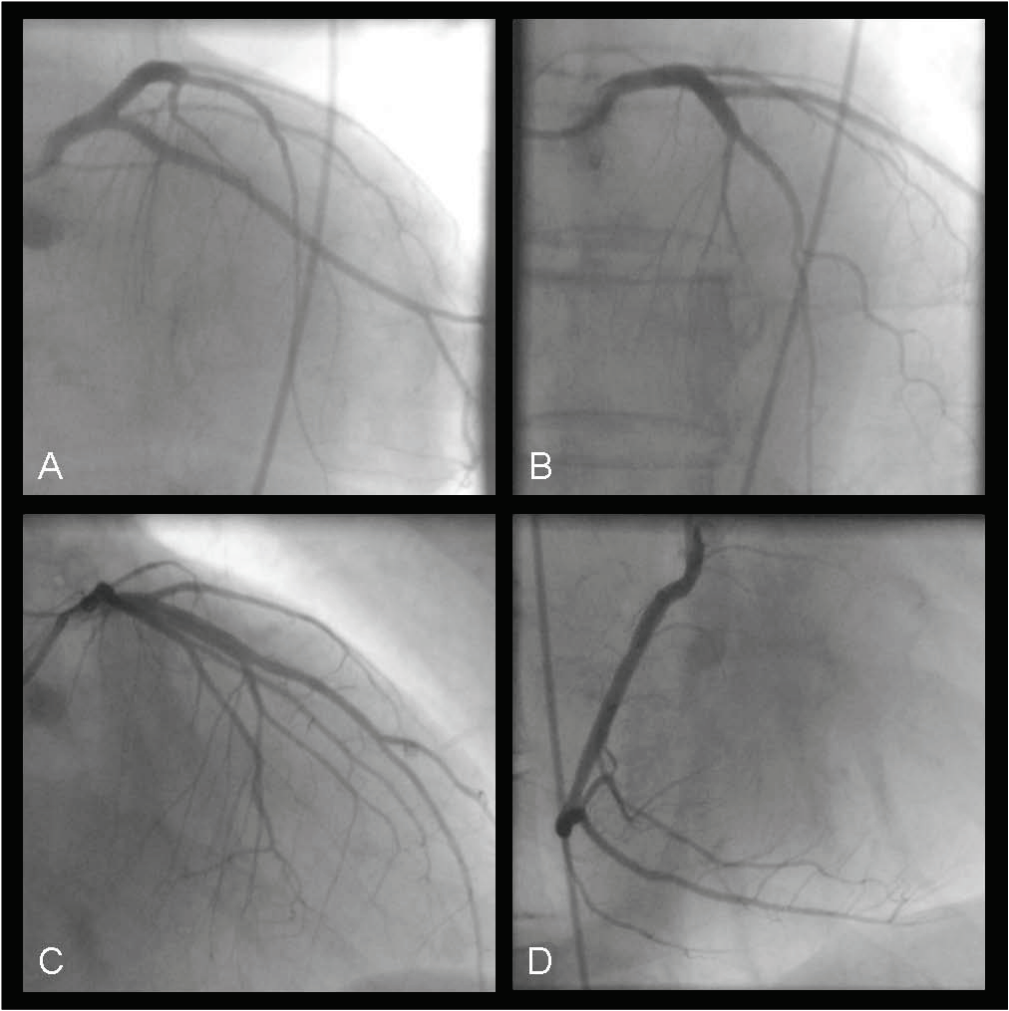
**Coronary angiography ruling out relevant coronary artery disease**. Left coronary artery: a left anterior oblique projection with caudal (A) and cranial (B) angulation and a right anterior oblique projection with cranial (C) angulation. (D): right anterior oblique projection of the right coronary artery.

**Figure 2:**
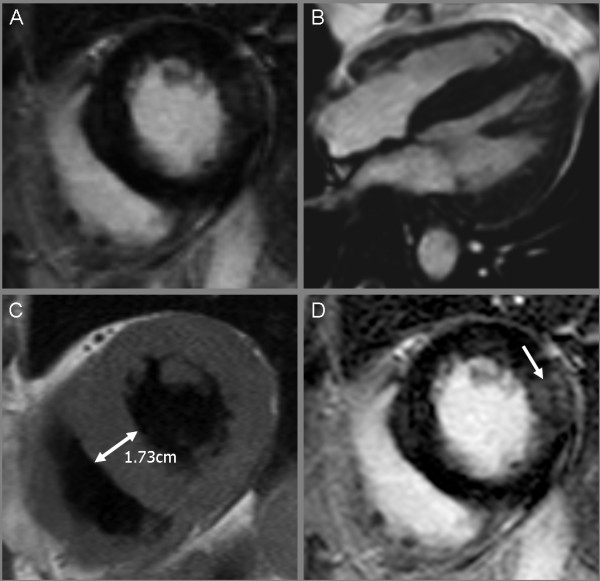
**Cardiac MRI showing a marked hypertrophic cardiomyopathy**. Balanced Fast Field Echo (bFFE) cine sequence, short axis view (A); 4 chamber view (B); a left ventricular hypertrophy with normal contractility was recognized. T1 weighted black blood Turbo spin echo sequence, short axis view (C); left ventricular hypertrophy is evident, specifically in the septal region. Corresponding view with transmural late Gadolinium enhancement (arrow) in the lateral wall using a T1 weighted inversion recovery 3D fast gradient echo sequence (D).

Two years after the onset of ERT, the patient described an episode of dyspnea during physical activity. The symptoms resolved at rest. A subsequent cTNI assessment revealed a value of 0.46 ng/ml (normal range <0.05 ng/ml). The patient was hospitalised and treated based on the regimen on an acute coronary syndrome. The actual coronary angiography revealed regular findings (Figure 1), while the cardiac MRI showed a remarkable left ventricular hypertrophy. Furthermore a late Gadolinium enhancement imaging indicated a focal parenchymal fibrosis, which is a common finding in FD patients with cardiac involvement (Figure 2). In line with findings depicted in the coronary angiography the electrocardiogram (ECG) did not show typical changes suggestive to an acute myocardial ischemia. A sinus rhythm (78/minutes), a normal cardiac axis and no pathological intervals were observed. The single obvious abnormality was an ST segment depression with a preterminal negative T wave in the leads I, II, aVL, aVF and V_3_-V_6_. In comparison, the ECG performed one year prior to the actual event revealed similar findings. On exercise ECG no cardiac symptoms occurred whereas the ECG morphology remained unchanged. The Holter ECG showed no relevant arrhythmias. Transthoracic echocardiography revealed left ventricular hypertrophy without any regions of wallhypokinesis or valves insufficiencies. On discharge a cTNI value of 0.67 ng/ml was measured. A therapy with aspirin 100 mg daily was established.

**Figure 3:**
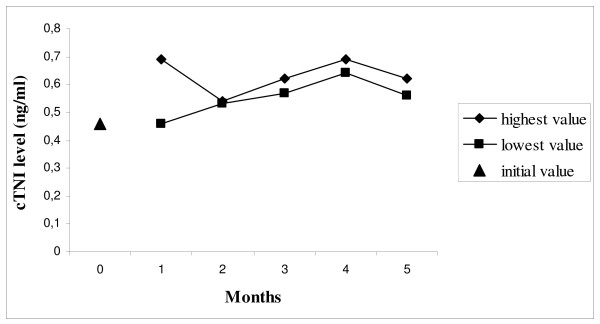
**Cardiac troponin I levels in ng/ml (highest and lowest values) within 5 months**.

Apart from slight palpitations after intensive exercise during the following 4 weeks, no further relevant cardiac symptoms occurred. The hospitalisation did not lead to an interruption of the enzyme replacement therapy. Over a period of 5 months the cTNI value was assessed at least twice monthly. Apart from slight fluctuations, the cTNI remained increased over this period (Figure 3). The ECG performed two months thereafter revealed findings consistent with previous examinations; in particular no inferior Q waves were evident in the subsequent ECG. A relevant deterioration of the renal function could not be observed following the diagnosis of FD, thus a significant renal insufficiency responsible for the elevated cTNI could be ruled out. Prior to enzyme replacement therapy the glomerular filtration rate (GFR) was 37.3 ml/min, the current measurement revealed a value of 53.4 ml/min (both values measured per 24-h urine/1.73 body surface area). The serum creatinine level did not exceed 1.1 mg/dl. As there was no evidence for an acute myocardial ischemia explaining the cTNI increase, the measurements of cTNI in short intervals was ceased.

## Discussion

The main finding in the present case is a persistent cTNI elevation in a FD patient with cardiac involvement. Left ventricular hypertrophy, which was evident in our patient, is one of the most frequent pathological cardiac finding in FD. In case of cardiac clinical manifestation it might result in cTNI elevation [4]. In contrast, the presented case demonstrates a persistence of increased cTNI level, even in symptom free intervals.

Since cTNI is considered highly specific for myocardial damage, usually prompting further cardiological investigation, including invasive methods such as a coronary angiography, our observation is of high clinical relevance [7,8]. As small vessel ischemic disease is the suspected cause of the myocardial damage in FD, a persistent elevation of cardiac biomarkers implicates a continuous silent ischemia [4]. Proving a persistent cTNI elevation in a symptom free interval, increased cTNI levels could be interpreted as a direct damage of myocytes due to the deposition of glycosphingolipids. Even though the latter mechanism appears likelier, the pathomechanism behind our observation requires further assessment. A cTNI elevation as a simple coincidental independent finding seams unlikely facing the marked cardiac changes in our patient. In case of evidence of a direct relationship between the cTNI elevation and degree of the cardiac damage in FD, a cTNI measurement would be of great value as a marker for the severity of cardiac involvement. It could also serve for long term monitoring in patients on ERT. Further investigations are therefore promptly needed to enable a better understanding of cTNI release and cardiac involvement in FD with important implications for the care of these patients.

Renal insufficiency, which is also a frequent manifestation in FD, needs to be taken into consideration when interpreting elevated cTNI values [17,18]. As demonstrated by Flisinski et al. in patients with chronic kidney disease who were treated with haemodialysis, cTNI values of 0.017 - 0.143 ng/ml occurred without any signs of acute myocardial damage; in patients with a GFR of < 53 ml/min cTNI levels did not exceed 0.23 ng/ml (99th percentile) [17]. In this context the reduced GFR in our patient might be of some relevance, however the increased values of 0.69 ng/ml can not solely be caused by renal insufficiency.

Elevation of cardiac biomarkers in the absence of an acute coronary syndrome has drawn more and more attention over the past years, indicating numerous conditions associated with cTNI release of unknown origin [9-15]. The evidence of a persistent and clinically silent cTNI elevation in FD would subsequently raise the question of the relevance of FD among patients with a cTNI release of unknown origin. Elevated cTNI levels might be of particular interest in patients with unclear renal insufficiency with respect to the diagnostic value in identifying patients with FD. In a high percentage of patients with FD renal and cardiac involvement are concomitant findings [19,20]. A possible consequence could be the implementation of cTNI assessment in the renal diagnostic algorithm for proving a cardiomyopathy, which potentially indicates involvement of FD. This would be of great value for an early detection of FD, prompting access to treatment in forms of ERT for this disease.

## Conclusions

In conclusion, our case report demonstrates a persistent cTNI release in the absence of clinical signs in a patient with cardiac involvement in FD. A further evaluation of cTNI in patients with FD appears mandatory, presumably serving as a beneficial long term parameter and providing new perspectives for screening strategies.

## Abbreviations

cTNI: cardiac troponin I; FD: Fabry disease; GFR: glomerular filtration rate.

## Consent

Written informed consent was obtained from the patient for publication of this case report and any accompanying images.

## Competing interests

The authors declare that they have no competing interests.

## Authors' contributions

TC, FA, WF, WO, NS, FR and SC carried out the data collection and drafted the manuscript. RA conducted the genetic analysis. All authors were intensively involved in the analysis and interpretation of the results and critically revised the manuscript for important intellectual content. All authors read and approved the final manuscript.

## Pre-publication history

The pre-publication history for this paper can be accessed here:

http://www.biomedcentral.com/1471-2261/11/6/prepub
